# Involvement of a Phage-Encoded Wzy Protein in the Polymerization of K127 Units To Form the Capsular Polysaccharide of Acinetobacter baumannii Isolate 36-1454

**DOI:** 10.1128/spectrum.01503-21

**Published:** 2022-04-27

**Authors:** Nikolay P. Arbatsky, Anastasiya A. Kasimova, Alexander S. Shashkov, Mikhail M. Shneider, Anastasiya V. Popova, Dmitry A. Shagin, Andrey A. Shelenkov, Yuliya V. Mikhailova, Yurii G. Yanushevich, Ruth M. Hall, Yuriy A. Knirel, Johanna J. Kenyon

**Affiliations:** a N. D. Zelinsky Institute of Organic Chemistry, Russian Academy of Sciences, Moscow, Russia; b M. M. Shemyakin & Y. A. Ovchinnikov Institute of Bioorganic Chemistry, Russian Academy of Sciences, Moscow, Russia; c Institute of Antimicrobial Chemotherapy, Smolensk State Medical University, Smolensk, Russia; d State Research Center for Applied Microbiology and Biotechnology, Obolensk, Moscow, Russia; e Central Scientific Research Institute of Epidemiology, Moscow, Russia; f School of Life and Environmental Sciences, University of Sydney, Sydney, Australia; g Centre for Immunology and Infection Control, School of Biomedical Sciences, Faculty of Health, Queensland University of Technology, Brisbane, Australia; Institut Pasteur

**Keywords:** *Acinetobacter baumannii*, capsular polysaccharide, K locus, K127, phage, Wzy polymerase

## Abstract

A comprehensive understanding of capsular polysaccharide (CPS) diversity is critical to implementation of phage therapy to treat panresistant Acinetobacter baumannii infections. Predictions from genome sequences can assist identification of the CPS type but can be complicated if genes outside the K locus (CPS biosynthesis gene cluster) are involved. Here, the CPS produced by A. baumannii clinical isolate 36-1454 carrying a novel K locus, KL127, was determined and compared to other CPSs. KL127 differs from KL128 in only two of the glycosyltransferase (*gtr*) genes. The K127 unit in 36-1454 CPS was the pentasaccharide β-d-Glc*p*-(1→6)-d-β-Gal*p*NAc-(1→6)-α-d-Gal*p*-(1→6)-β-d-Glс*p*-(1→3)-β-d-Gal*p*NAc in which d-Glc*p* at position 4 replaces d-Gal*p* in K128, and the glycosyltransferases encoded by the different *gtr* genes form the surrounding linkages. However, although the KL127 and KL128 gene clusters encode nearly identical Wzy polymerases, the linkages between K units that form the CPS chains are different, i.e., β-d-Gal*p*NAc-(1→3)-d-Gal*p* in 36-1454 (K127) and β-*d-*Gal*p*NAc-(1→4)-d-Gal*p* in KZ-1093 (K128). The linkage between K127 units in 36-1454 is the same as the K-unit linkage in five known CPS structures, and a gene encoding a Wzy protein related to the Wzy of the corresponding K loci was found encoded in a prophage genome in the 36-1454 chromosome. Closely related Wzy proteins were encoded in unrelated phage in available KL127-carrying genomes. However, a clinical isolate, KZ-1257, carrying KL127 but not the prophage was found, and K127 units in the KZ-1257 CPS were β-d-Gal*p*NAc-(1→4)-d-Gal*p* linked, confirming that Wzy_KL127_ forms this linkage and thus that the phage-encoded Wzy_Ph1_ forms the β-d-Gal*p*NAc-(1→3)-d-Gal*p* linkage in 36-1454.

**IMPORTANCE** Bacteriophage therapy is an attractive innovative treatment for infections caused by extensively drug resistant Acinetobacter baumannii, for which there are few effective antibiotic treatments remaining. Capsular polysaccharide (CPS) is a primary receptor for many lytic bacteriophages, and thus knowledge of the chemical structures of CPS produced by the species will underpin the identification of suitable phages for therapeutic cocktails. However, recent research has shown that some isolates carry additional genes outside of the CPS biosynthesis K locus, which can modify the CPS structure. These changes can subsequently alter phage receptor sites and may be a method utilized for natural phage resistance. Hence, it is critical to understand the genetics that drive CPS synthesis and the extent to which genes outside of the K locus can affect the CPS structure.

## INTRODUCTION

Bacteriophages are currently being investigated for use in novel therapies against the notoriously antibiotic-resistant bacterial pathogen, Acinetobacter baumannii (1–4). A major receptor for specific phage is the capsular polysaccharide (CPS) layer on the A. baumannii cell surface (5–8). The CPS is a high-molecular-weight carbohydrate polymer which is comprised of oligosaccharide units known as K units that are linked together by a Wzy polymerase. However, different isolates have different CPS structures (9), and phages that enter via the CPS can encode a depolymerase that recognizes only one or a few specific CPS structures (5, 10). Hence, the development of therapeutic bacteriophage cocktails is complicated by CPS diversity. To enable the rapid identification of suitable phages using whole-genome sequences of relevant isolates, it is important to understand the relationship between the genetics that drive synthesis of each CPS type and the variety in CPS structures produced by this species.

In A. baumannii, the majority of CPS biosynthesis genes are clustered at the chromosomal K locus, which is located between the *fkpA* and *lldP* genes (9, 11). However, more than 140 different gene clusters (KL) have been identified at this chromosomal location (9, 12), and each cluster is assigned a unique KL number. Variation in the genes present at the K locus leads to extensive variation in the sugar composition and structure of the CPS in otherwise closely related isolates carrying different KL in both local and global populations (13). For example, available sequences of the two dominant clonal complexes that include most of the difficult-to-treat extensively or panresistant isolates, namely, global clones 1 and 2, were found to include 12 or 30 different KL, respectively (9), and where structures are available, the K units that make up the CPSs differ in sugar composition and linkages between the sugars. The linkage between K units in the CPS polymer can also differ, and cases where identical K units are made but are linked in different ways in different isolates using different Wzy polymerases are known (14–16).

Although the KL type can be identified in genomic sequences using the recently developed Kaptive tool with the A. baumannii database (9), CPS structure may also be affected by genes carried by genomic islands (17, 18) or located in prophage genomes (16) found integrated elsewhere in the chromosome. Therefore, it is crucial to continue to determine CPS structures in order to expand the ability to predict structure from whole-genome sequences and to examine the entire genome sequence for CPS biosynthesis genes needed to generate the structure produced by the isolate.

In this work, we describe a new CPS structure produced by A. baumannii isolate 36-1454 and investigate the genetics that drive the synthesis of this structure. The role of a phage-encoded K-unit polymerase, found by examining the whole-genome sequence of 36-1454, determineing the linkage between K units was established.

## RESULTS

### Genome sequence of A. baumannii 36-1454 includes a novel gene cluster at the K locus.

Genomic material from A. baumannii clinical isolate 36-1454, recovered in Moscow, Russia, in 2013, was extracted and sequenced using an Illumina MiSeq platform. The draft genome sequence was assembled from short read data and deposited in NCBI under accession number JAHTLH000000000.1. The 36-1454 genome sequence was found to belong to ST448 in the A. baumannii Institut Pasteur multilocus sequence typing (MLST) scheme and to ST1174 in the Oxford MLST scheme.

**FIG 1:**
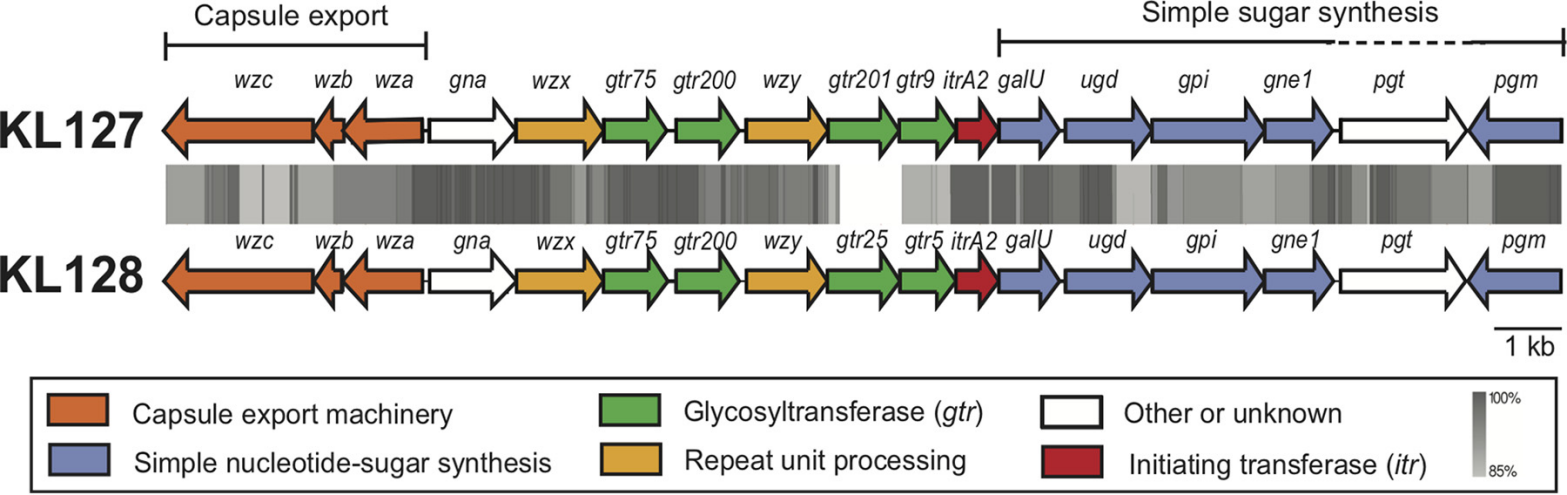
A. baumannii KL127 and KL128 capsule biosynthesis gene clusters. Genes are color coded according to the functions of their encoded products, and the color scheme is shown below the diagram. Gray shading represents nucleotide sequence identity generated by tblastx comparisons with Easyfig (36), and the scale is shown below the diagram. The figure is drawn to scale based on sequences and annotations from GenBank accession numbers MK399427.1 (KL127) and MK399428.1 (KL128).

The K locus was found to contain a novel CPS biosynthesis gene cluster, which was named KL127. The fully annotated sequence can be found in GenBank under accession number MK399427.1. As for most A. baumannii CPS biosynthesis gene clusters (9), KL127 (Fig. 1) includes genes for capsule export (*wza-wzb-wzc*), synthesis of simple sugar substrates (*galU-pgm*), K-unit translocation across the inner membrane (*wzx*), and K-unit polymerization (*wzy*). It also includes a gene for an initiating transferase (Itr) to begin K-unit synthesis and four glycosyltransferase genes (*gtr*) to link sugars together to form complete K units prior to polymerization by a specific Wzy and export of the CPS polymer to the cell surface.

### KL127 and KL128 capsule biosynthesis gene clusters.

The KL127 gene cluster closely resembles the KL128 gene cluster (GenBank accession number MK399428.1) from A. baumannii KZ-1093 described previously (19), sharing 96.46% nucleotide sequence identity over 18,816 bp of the 20,713-bp locus (Fig. 1). KL127 and KL128 both include the *itrA2* gene for a d-Gal*p*NAc-1-phosphate transferase and the *gtr75* and *gtr200* genes, and the roles of the encoded glycosyltransferases in the synthesis of the K128 CPS structure were deduced previously (19). Gtr75 forms a β-d-Glc*p*-(1→6)-d-Gal*p*NAc linkage, whereas Gtr200 was shown to be responsible for β-d-Gal*p*NAc-(1→6)-d-Gal*p*. Thus, the same linkages are expected in the K127 unit.

Similarly, KL127 and KL128 encode Wzy proteins that are 97.7% identical. Previously, Wzy_KL128_ was assigned to formation of the β-d-Gal*p*NAc-(1→4)-d-Gal*p* linkage between K128 units (19), and the protein sequence shared 53.9% identity with Wzy_KL27_ encoded by the A. baumannii KL27 gene cluster which has unambiguously been shown to form a β-d-Gal*p*NAc-(1→4)-d-Gal*p* linkage between units in the K27 CPS (14). Therefore, as the *wzy* genes in KL127 and KL128 are nearly identical, a β-d-Gal*p*NAc-(1→4)-d-Gal*p* linkage is also expected between the K127 units as in K128.

The difference between the KL127 and KL128 gene clusters lies in a small region that includes two of the four glycosyltransferase genes, where *gtr201-gtr9* in KL127 replaces *gtr25-gtr5* in KL128. Gtr9 has been described previously for the synthesis of the A. baumannii K37 CPS and is responsible for the β-(1→3) linkage of d-Glc*p* to the d-Gal*p*NAc initiating sugar of the K37 unit (20, 21). However, Gtr9 is related to Gtr5 (Fig. 1), which links d-Gal*p* to a d-Gal*p*NAc initiating sugar (22, 23), a linkage also seen in K128. Although the *gtr201* glycosyltransferase gene is novel (i.e., <85% identical to previously assigned Gtrs), it predicts a product (GenPept accession number QBM04716.1) that shares 75% amino acid sequence identity with Gtr77_KL37_, also encoded by the KL37 gene cluster (GenBank accession number KX712115.1). Gtr77_KL37_ catalyzes the transfer of d-Gal*p* to a d-Glc*p* residue via a α-(1→6) linkage in the K37 CPS. Therefore, K127 is predicted to share structural similarities with both K128 and K37.

### Elucidation of the 36-1454 CPS structure.

**FIG 2:**
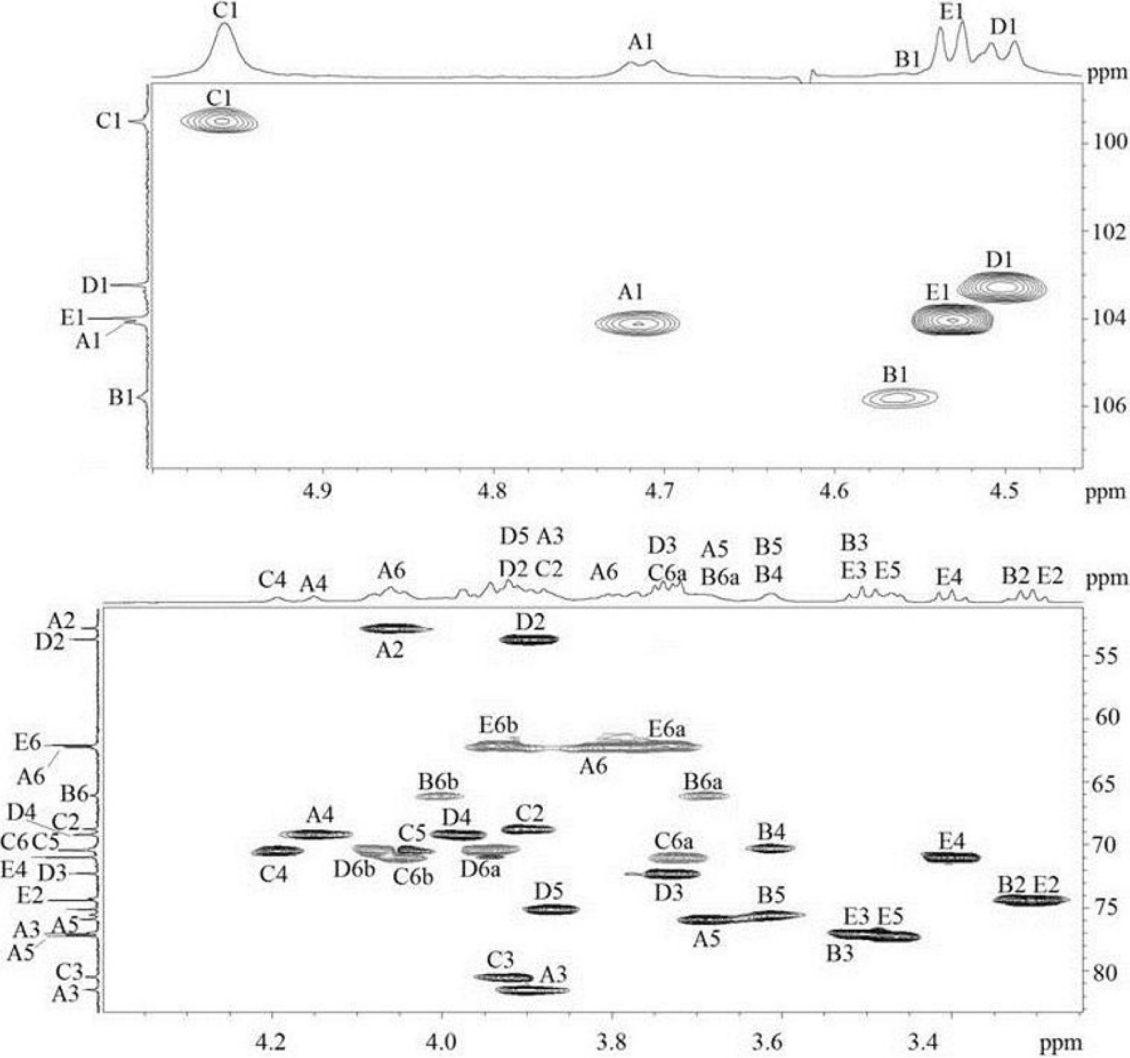
Parts of a two-dimensional ^1^H,^13^C HSQC spectrum of the CPS of A. baumannii 36-1454. The corresponding parts of the ^1^H and ^13^C NMR spectra are shown along the horizontal and vertical axes, respectively. Numbers refer to H/C pairs in sugar residues denoted by letters as indicated in Table 1.

A CPS preparation was isolated from cells of A. baumannii 36-1454. Sugar analysis using a sugar analyzer after full acid hydrolysis of the CPSs revealed the presence of Glc, Gal, and GalNAc at a ratio of ~0.8:1.3:1.7. The CPS structure was established by NMR spectroscopy, including two-dimensional ^1^H,^1^H correlation spectroscopy (COSY), ^1^H,^1^H total correlation spectroscopy (TOCSY), ^1^H,^1^H rotating-frame nuclear Overhauser effect spectroscopy (ROESY), ^1^H,^13^C heteronuclear single quantum coherence (HSQC) (Fig. 2), and ^1^H,^13^C heteronuclear multiple-bond correlation (HMBC) experiments. The assigned ^1^H and ^13^C NMR chemical shifts of the CPSs are tabulated in Table 1.

**TABLE 1:** ^1^H and ^13^C NMR chemical shifts

Sugar residue	δ (ppm)^*a*^					
	C-1 *H-1*	C-2 *H-2*	C-3 *H-3*	C-4 *H-4*	C-5 *H-5*	C-6 *H-6 (6a, 6b)*
K127-Wzy_Ph1_ CPS of A. baumannii 36-1454						
→3)-β-d-Gal*p*NAc-(1→ A	104.1 *4.71*	52.7 *4.06*	81.4 *3.89*	69.1 *4.15*	75.8 *3.69*	62.2 *3.77, 3.80*
→6)-β-d-Glc*p*-(1→ B	105.8 *4.56*	74.2 *3.32*	76.9 *3.52*	70.2 *3.61*	75.5 *3.61*	66.0 *3.69, 4.00*
→3,6)-α-d-Gal*p*-(1→ C	99.5 *4.96*	68.7 *3.90*	80.4 *3.91*	70.4 *4.19*	70.4 *4.04*	70.9 3*.72, 4.05*
→6)-β-d-Gal*p*NAc-(1→ D	103.2 *4.50*	53.6 *3.89*	72.2 *3.73*	69.2 *3.97*	75.0 *3.87*	70.3 *3.94, 4.07*
β-d-Glc*p*-(1→ E	104.0 *4.53*	74.3 *3.31*	76.9 *3.50*	70.9 *3.39*	77.2 *3.47*	62.0 *3.73, 3.93*

Glycoside from the K127-Wzy_Ph1_ CPS						
β-d-Gal*p*NAc-(1→ A	104.2 *4.65*	53.7 *3.94*	71.9 *3.76*	68.9 *3.95*	76.0 *3.68*	62.1 *3.71,3.78*
→3)-α-d-Gal*p*-(1→ C	99.7 *4.94*	68.5 *3.89*	80.2 *3.96*	70.2 *4.21*	71.6 *3.96*	62.2 *3.73,3.74*
→1)-glycerol B′	69.6 *3.58, 3.76*	69.5 *3.95*	71.2 *3.65, 3.68*			

a ^1^H NMR chemical shifts are italicized. Chemical shifts for the *N*-acetyl groups are as follows: δ_H_, 2.03 to 2.07; δ_C_, 23.7 to 24.1 (CH_3_) and 175.7 to 176.3 (CO).

Nuclear magnetic resonance (NMR) analysis revealed spin systems for five monosaccharide residues (units A to E), all being in the pyranose form (Table 1). In the ^1^H,^1^H TOCSY spectrum, there were correlations for H-1 with H-2,3,4 for sugars having the *galacto* configuration (Gal and GalNAc) and with H-2,3,4,5 for Glc. The signals within each spin system were assigned using the ^1^H,^1^H COSY spectrum, and those for H-5 and H-6 of Gal and GalNAc were found by H-4/H-5 correlations in the ^1^H,^1^H ROESY spectrum and H-5/H-6 correlations in the ^1^H,^1^H COSY spectrum. Relatively large *J*_1,2_ coupling constants of 7 to 8 Hz indicated that all monosaccharide residues are β-linked, except for the Gal residue (unit C), which is α-linked, as judged by a relatively low ^3^*J*_1,2_ coupling constant of <3 Hz.

Downfield displacements by 6 to 10 ppm of the signals for the linkage carbons, relative to their positions in the spectra of the corresponding nonsubstituted monosaccharides (24), showed that the CPS is branched and defined the glycosylation pattern in the K unit. In the ^1^H,^1^H ROESY spectrum, there were correlations for the anomeric proton of each monosaccharide with protons at the linkage carbons of the neighboring sugar residue, which confirmed the positions of substitution and defined the sequence of the monosaccharides in the K unit.

**FIG 3:**
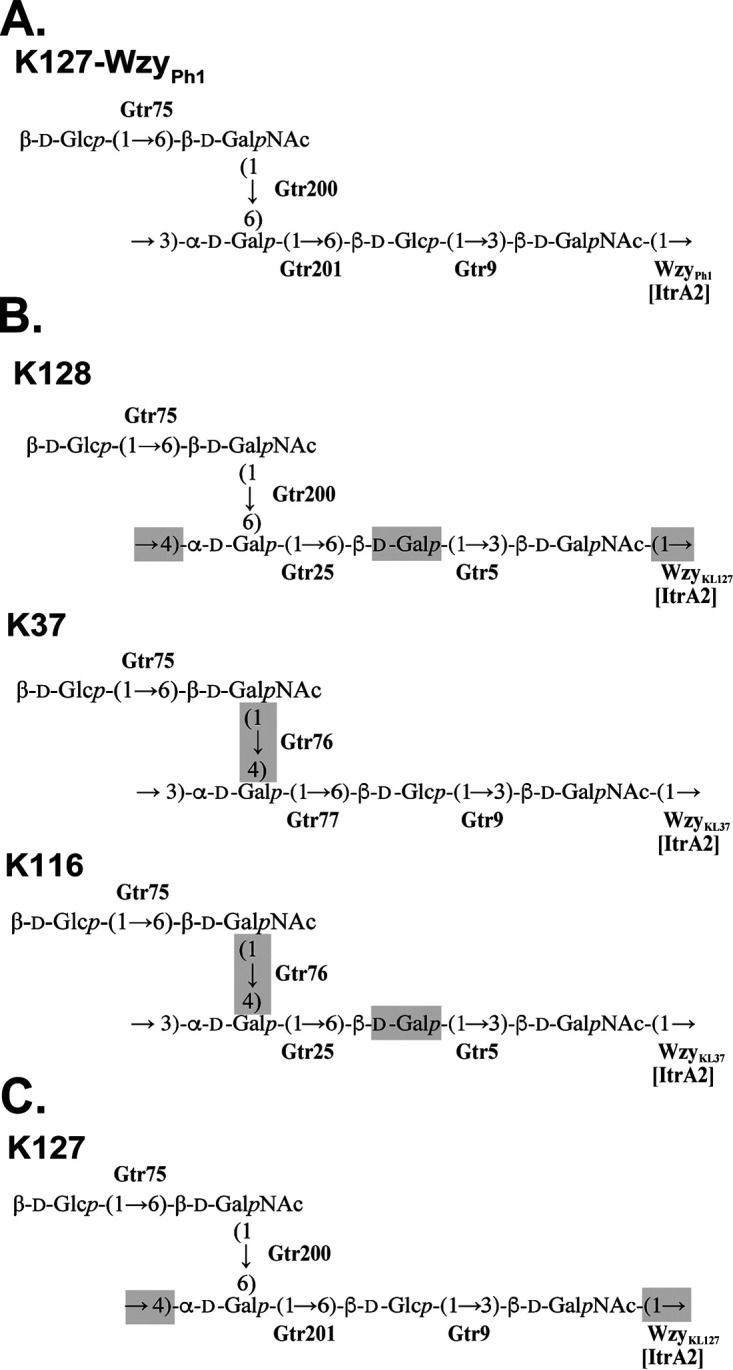
(A) Structure of the K127-Wzy_Ph1_ CPS from A. baumannii 36-1454 (this study). (B and C) Structures of the related CPSs of A. baumannii K128 (19) and K37 and K116 (21) (B) and of A. baumannii KZ-1257 (this study) (C). Differences from K127-Wzy_Ph1_ are highlighted by gray boxes. Enzymes are indicated in bold next to the linkage they are predicted to catalyze.

The structure of the CPS from isolate 36-1454 thus established (Fig. 3A) was confirmed by Smith degradation, which cleaved the β-d-Glc (units B and E) and β-d-GalNAc (unit D) residues to give a β-d-Gal*p*NAc-(1→3)-α-d-Gal*p*-(1→1)-Gro oligosaccharide glycoside with glycerol (Gro) as aglycon. Its structure was established by NMR spectroscopy as described above (for the assigned ^1^H and ^13^C NMR chemical shifts of the glycoside, see Table 1).

### Correlation of KL127 genes with the CPS structure from 36-1454.

**FIG 4:**
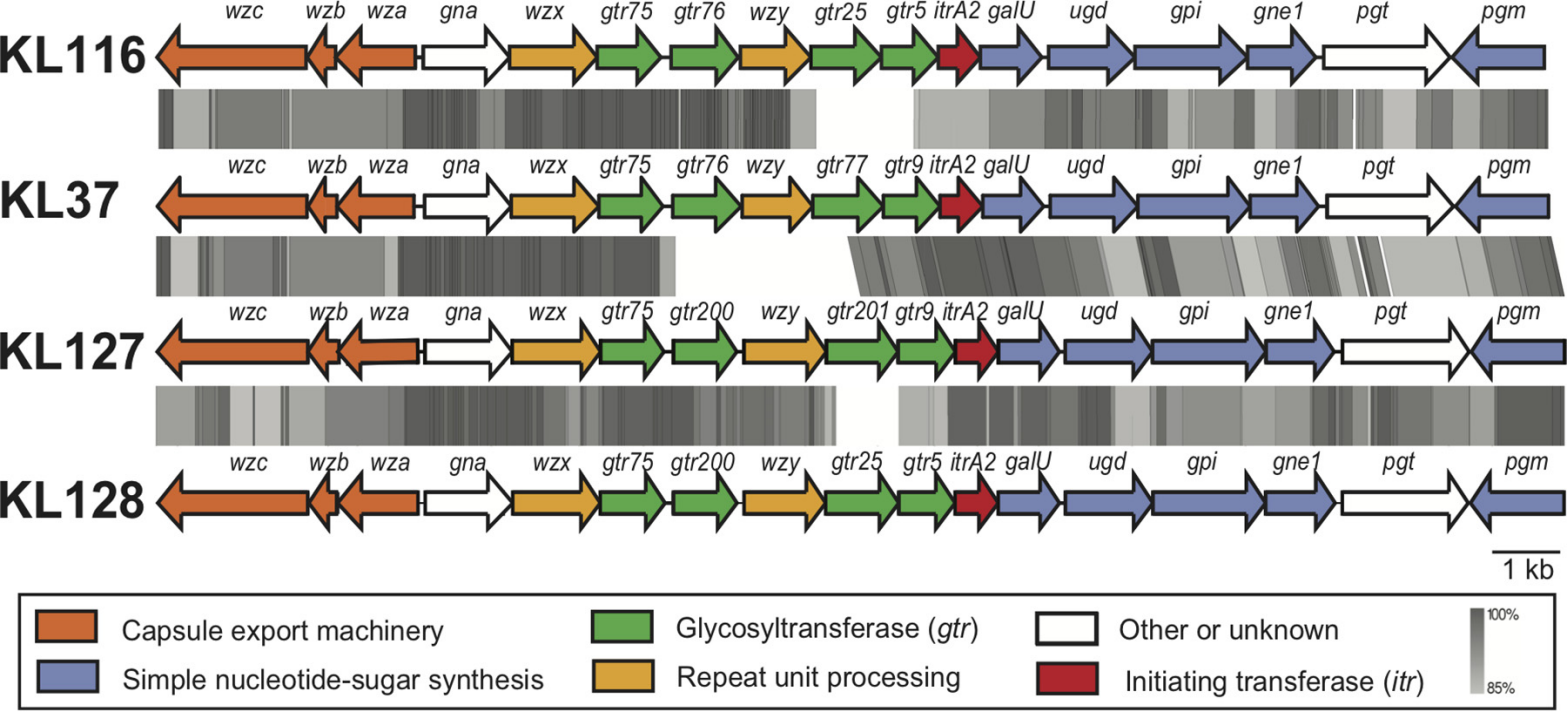
Related A. baumannii KL gene clusters compared with KL127. Colors represent functional groups of encoded gene products, and the color scheme is shown below the diagram. Gray shading indicates nucleotide sequence identity generated by tblastx with Easyfig (36), with the scale shown below the diagram. The figure is drawn to scale based on sequences from GenBank accession numbers MK399427.1 (KL127), KX712115.1 (KL37), MK399425.1 (KL116), and MK399428.1 (KL128).

The elucidated CPS structure includes a β-d-Glc*p*-(1→6)-d-Gal*p*NAc disaccharide side chain that is β-(1→6) linked to an α-d-Gal*p*-(1→6)-β-d-Glc*p*-(1→3)-β-d-Gal*p*NAc trisaccharide main chain. The internal linkages in the main chain are identical to those in the K37 main chain (Fig. 3B), confirming the predicted roles of both Gtr201 and Gtr9 in K127 (see above). The β-d-Glc*p*-(1→6)-β-d-Gal*p*NAc side branch in the K127 unit is also found in both K37 and K128 and the closely related A. baumannii K116 structure (21). Each of the corresponding gene clusters includes a *gtr75* gene (Fig. 4), indicating that Gtr75 catalyzes the formation of this linkage as predicted previously (19, 21). The remaining β-d-Gal*p*NAc-(1→6)-d-Gal*p* linkage in the K127 unit that links the side branch to the main chain is shared only with K128, and as *gtr200* is found in both KL127 and KL128, Gtr200 would form this linkage.

### Linkage between the K127 units.

Although the Wzy proteins encoded by KL127 and KL128 (GenPept accession numbers QBM04715.1 and QBM04739.1, respectively) are 97.7% identical (8 of 348 amino acids differ, N92S, S95A, I98L, V133A, F150C, S208C, C237V, and F254V), the linkage between K units in the 36-1454 and KZ-1093 CPSs were unexpectedly different. Wzy_KL128_ forms a β-d-Gal*p*NAc-(1→4)-d-Gal*p* linkage between K128 units in KZ-1093 (19), whereas a β-d-Gal*p*NAc-(1→3)-d-Gal*p* linkage joins K127 units in the 36-1454 CPS (Fig. 3A). Interestingly, the K127 linkage is identical to that found between K units in the K37 and K116 CPSs (Fig. 3B), and the KL37 and KL116 gene clusters (Fig. 4) encode closely related Wzy proteins (95% identical), designated Wzy_KL37_ (GenPept accession number AQQ74322.1) and Wzy_KL116_ (QBM04667.1), respectively. However, Wzy_KL37_ and Wzy_KL116_ share no significant identity with Wzy_KL127_, although all are members of protein family (Pfam) EpsG (PF14897). Therefore, the possibility that an alternate Wzy that shares similarity with Wzy_KL37/KL116_ is encoded elsewhere in the genome was investigated.

### An additional *wzy* gene in the 36-1454 genome.

As the linkage between K127 units is identical to the Wzy linkage previously characterized for both the K37 and K116 structures, all coding sequences in the 36-1454 draft genome sequence were translated (*n* = 3,833) and initially searched against Wzy_KL37_ and Wzy_KL116_ sequences using BLASTp. This search identified a protein (GenPept accession number MBV6766733.1) encoded outside the K locus that is 38 to 40% identical to Wzy_KL37_ and Wzy_KL116_ (Table 2). All translated coding sequences were also searched against an in-house database of concatenated A. baumannii Wzy sequences from strains with known CPS structures where the Wzy had been assigned to a specific linkage between K units in previous studies. A further three Wzy sequences from A. baumannii were found to share 32 to 36% identity with the same candidate protein, and all three had previously been assigned to β-d-Gal*p*NAc-(1→3)-d-Gal*p* linkages (see Table 2). These three Wzy proteins also share 31 to 54% identity with Wzy_KL37_ and Wzy_KL116_. Therefore, matches to the candidate protein were considered significant, providing support for the conclusion that this protein forms the β-d-Gal*p*NAc-(1→3)-d-Gal*p* linkage between K127 units. Equivalent searches of the draft genome sequence of A. baumannii isolate KZ-1093 (NCBI WGS accession number JAJAWC000000000.1; KL128) yielded no additional Wzy homologues. Likewise, searches of the strains used to determine the K3 and K37 structures did not reveal additional potential *wzy* genes.

**TABLE 2:** A. baumannii Wzy proteins sharing significant homology with Wzy_Ph1_ encoded by the 36-1454 genome

Wzy protein	Amino acid sequence identity to Wzy_Ph1_ (%)	Alignment coverage (%)	Linkage catalyzed by Wzy protein	Reference(s)
Wzy_K116_	39.6	100	β-d-Gal*p*NAc-(1→3)-d-Gal*p*	21
Wzy_K37_	37.9	99	β-d-Gal*p*NAc-(1→3)-d-Gal*p*	20, 21
Wzy_K22_	36.4	98	β-d-Gal*p*NAc-(1→3)-d-Gal*p*	20
Wzy_K3_	34.5	99	β-d-Gal*p*NAc-(1→3)-d-Gal*p*	20
Wzy_K52_	31.9	97	β-d-Gal*p*NAc-(1→3)-d-Gal*p*	37

### The additional Wzy encoded by strain 36-1454 is phage encoded.

The specific contig of the 36-1454 draft genome sequence that contains the second *wzy* gene was examined in order to identify the genetic context of the candidate gene. The sequence was submitted to the PHASTER tool, which identified a 41.6-kb region encoding 29 proteins of phage origin with terminal *attL* and *attR* sites at either end and a tyrosine-type recombinase/integrase (GenPept accession number MBV6766697.1) encoded by a gene adjacent to the *attL* site. The candidate *wzy* gene was found within this phage sequence, suggesting that it was acquired via bacteriophage transfer and subsequent integration of the phage genome into the chromosome. Neither the prophage nor the candidate *wzy* gene sequence could be found in the draft genome sequence of A. baumannii isolate KZ-1093 that produces the related K128 CPS (19). As it appears that this protein has a role in the synthesis of the CPS produced by 36-1454, the gene was designated *wzy_Ph1._*

### Distribution of *wzy_Ph1_* and KL127 in publicly available Acinetobacter genomes.

Over 9,000 A. baumannii genomes, available in the Whole Genome Shotgun database (as of 22 July 2021), were downloaded and assessed to identify any further instances of the *wzy_Ph1_* gene in the species. The *wzy_Ph1_* sequence (100% coverage, 97% identity) was found in two further isolates: UBA3169 (WGS accession number DEYB01000000) recovered from an environmental sample (wood) in New York City, USA, and TUM15229 (WGS accession number BKLU01000007.1) recovered from a clinical sample (sputum) in Kanagawa, Japan, in 2013. These isolates have different sequence types (ST) (Table 3) and hence are not of the same lineage as 36-1454 or of one another, suggesting a sporadic pattern. However, the draft genome sequences of UBA3169 and TUM15229 were also found to include the KL127 gene cluster at the K locus. KL127 was not found in any other A. baumannii genomes that were available in the NCBI WGS database at the time of download, indicating a cooccurrence of KL127 with *wzy_Ph1_* in A. baumannii.

**TABLE 3:** A. baumannii genome sequences with phage genomes carrying the *wzy_Ph1_* gene

Strain	K locus type	City/country of isolation	ST^IP^/ST^Ox^^*c*^	GenBank accession no.	PHASTER match	Coordinates of phage genome	Coordinates of *wzy_Ph1_*
36-1454	KL127	Smolensk, Russia	ST448/ST1174	JAHTLH010000003.1	PHAGE_Bordet_BPP_1_NC_005357(14) [incomplete]	92161–133951	131401–132444
UBA3169	KL127	New York City, USA	ST428/ST936	DEYB01000056.1	PHAGE_Bordet_BPP_1_NC_005357(13) [questionable]	6886–48671	9593–10636
TUM15229	KL127	Kanagawa, Japan	ST193/ST741	BKLU01000002.1	PHAGE_Ralsto_RSK1_NC_022915(2) [incomplete]	63302–82581	75604–76645
AC1631^*a*^	KL127-like^*b*^	Malaysia		JAGSNH010000003.1	PHAGE_Ralsto_RSK1_NC_022915(2) [incomplete]	1–7207	3178–4221

a Acinetobacter nosocomialis.

b K locus designations in the current nomenclature system are A. baumannii only.

c ST^IP^, Institut Pasteur MLST; ST^Ox^, Oxford MLST.

A search for *wzy_Ph1_* outside of A. baumannii identified a single sequence from Acinetobacter nosocomialis strain AC1631 recovered in Malaysia in 2016. The encoded product (GenPept accession number MBR7749371.1) was found to share 99% amino acid sequence identity with Wzy_Ph1_ from A. baumannii 36-1454. Interestingly, the K locus in the genome of A. nosocomialis AC1631 (NCBI WGS accession number JAGSNH010000012.1) was found to include a region with 100% coverage and 94% sequence identity to the KL127 gene cluster from A. baumannii 36-1454, again suggesting the co-occurrence of a KL127 sequence with *wzy_Ph1_* in other species.

### The *wzy_Ph1_* gene is always in prophage.

**FIG 5:**
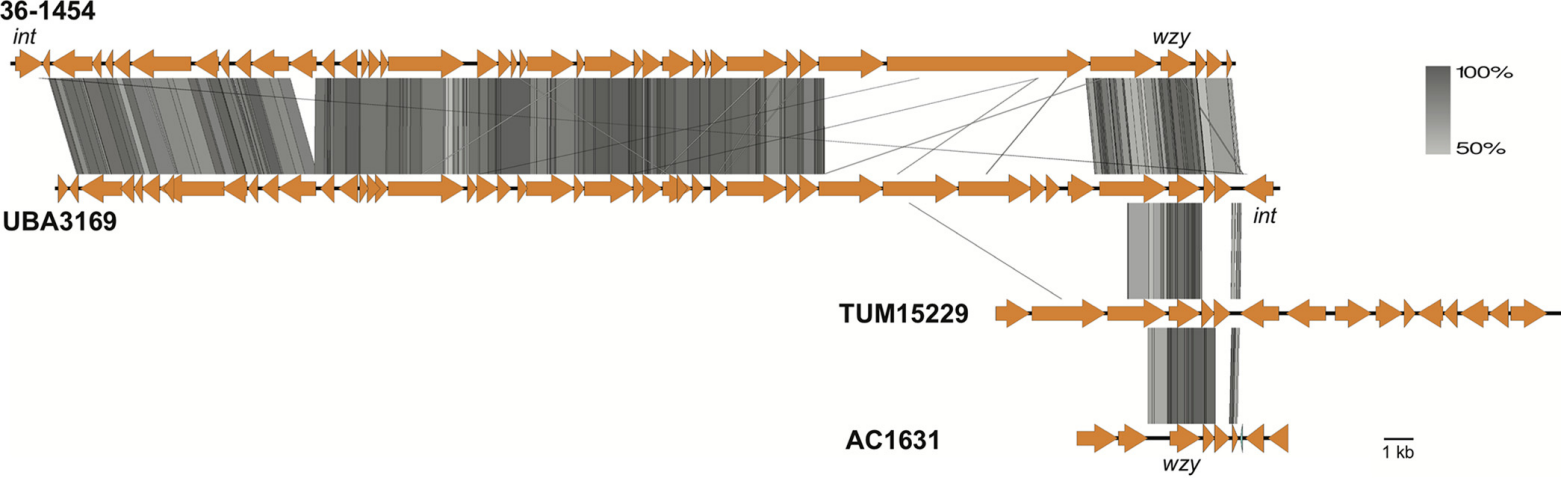
Comparison of prophage sequences carrying *wzy_Ph1_*. Strain names are shown to the left of the sequences. Orange arrows indicate open reading frames, and the location of *wzy_Ph1_* is indicated. Gray shading indicates sequence identity generated by tblastx with Easyfig (36), with the scale shown at the top right.

The genetic context of *wzy_Ph1_* was also examined for both UBA3169 and TUM15229 isolates, and the gene was again located within prophage sequence in both genomes (Table 3). However, the prophage sequence carrying *wzy_Ph1_* was not identical in these two strains or to the prophage found in the 36-1454 genome (Fig. 5), suggesting that several different phages have independently acquired *wzy_Ph1_*. Similarly, the contig containing the A. nosocomialis *wzy* gene (WGS accession number JAGSNH010000003.1) was subjected to PHASTER analysis. PHASTER revealed hits within the first 7,207 bases to the same prophage sequence carrying *wzy_Ph1_* found in the TUM15229 genome (Table 2), and as *wzy_Ph1_* is located within this span at base positions 3178 to 4221, the gene is also found in the prophage sequence in A. nosocomialis. However, the TUM15229 and AC1631 prophage sequences are not the same (Fig. 5).

### Identification of an A. baumannii isolate with KL127 but not *wzy_Ph1_*.

As A. baumannii isolate 36-1454 was obtained from the Institute of Antimicrobial Chemotherapy, Smolensk, collection of clinical isolates (https://snpt.antibiotic.ru/aba/#/), the sequences available in-house of further isolates from this collection were examined for further instances of KL127. This revealed a second A. baumannii clinical isolate, KZ-1257, recovered in Kazakhstan in 2016 that carried the KL127 gene cluster. The draft genome assembly was uploaded to NCBI and is available under accession number JALDNC000000000. Unlike 36-1454 and the other two KL127 isolates identified above, the KZ-1257 sequence belongs to ST498 in the Institut Pasteur MLST scheme and has no known ST in the Oxford scheme, indicating a different ancestral origin. While this genome included the KL127 gene cluster, the prophage carrying the additional *wzy* gene identified above was not present.

The structure of the CPS produced by KZ-1257 was determined as described above for 36-1454 (data not shown), and the K unit was found to be identical to the K127 unit from 36-1454. However, the linkage between K units was β-d-Gal*p*NAc-(1→4)-d-Gal*p* (Fig. 3C), as in KZ-1093 (KL128) and as expected if the Wzy_KL127_ produced by the gene in KL127 formed the linkage.

## DISCUSSION

K units produced by A. baumannii 36-1454 have a structure closely related to that of a number of other branched A. baumannii CPSs with pentasaccharide K units made up of neutral sugars, namely, K128, K37, and K116. These structures have a similar monosaccharide composition and the same topology (Fig. 3), although they differ in positions of substitution of the α-d-Gal residue (unit C) at the branching point (3,4, 4,6, or 4,6) and the nature of unit B in the main chain of the CPSs, which is either β-d-Gal or β-d-Glс. In general, these structural differences correlate with changes in the sequences of specific *gtr* or *wzy* genes at the K locus. We have previously shown that such small gene replacements in otherwise closely related gene clusters found at the A. baumannii K locus can alter the structure of the CPS produced and that these genetic relationships can assist with establishing the linkages formed by the encoded glycosyltransferases or Wzy polymerases (14–23, 25–29). However, while KL127 and KL128 were found to share very closely related *wzy* genes, the linkages between K units in the 36-1454 CPS and the K128 CPS of KZ-1093 are different.

It seemed unlikely that the few amino acid differences could have altered the linkage specificity between the Wzy proteins encoded by KL127 and KL128, and the finding of an alternate Wzy encoded in a phage sequence elsewhere in the 36-1454 genome can more readily explain the difference in the Wzy-catalyzed linkage between K127 units in 36-1454 and K128 units in KZ-1093. Moreover, no additional Wzy proteins were found encoded in the KZ-1093 (KL128) genome. This conclusion is corroborated by the finding that the expected linkage between K127 units was detected in another isolate that includes KL127 but not the prophage. In addition, a mutant in which the *wzy_Ph1_* gene had been deleted (and replaced by a kanamycin resistance determinant) produced CPS in which the K127 units were linked via a β-d-Gal*p*NAc-(1→4)-d-Gal*p* linkage (unpublished observations). Therefore, the CPS of 36-1454 elucidated in this study should be considered a phage-modified variant of the true K127 CPS structure that is found in KZ-1257. Hence, we named the CPS type of 36-1454 K127-Wzy_Ph1_.

Currently, it is unclear why the KL127-encoded Wzy would not contribute to polymerization of the K127 units, as no sequence insertions or deletions were identified in the *wzy* gene in KL127 of 36-1454, and a knockout of the *wzy_Ph1_* gene restored theβ-d-Gal*p*NAc-(1→4)-d-Gal*p* linkage. It is also unclear why the phage-encoded Wzy would override its function. However, it is possible that a yet unidentified phage-encoded factor may suppress the activity of the KL-encoded Wzy or that the *wzy_Ph1_* gene is better expressed, and further work will be needed to establish this. The coexistence of two Wzy enzymes in the same strain has been previously reported for Pseudomonas aeruginosa (30) and Salmonella enterica (31), and in the strains examined, only one polymerase was found to be functional. Both studies also proposed that the alternate *wzy* gene may be of phage origin. That the *wzy_Ph1_* gene was otherwise found only in available Acinetobacter genomes carrying KL127 suggests that Wzy_Ph1_ may be able only to form the linkage between a specific type of K unit represented by K127.

Relatively little attention has been paid to the diversity and specificity of Acinetobacter Wzy proteins, and structural data are needed to identify the linkage formed by each one. However, several homologous Wzy proteins encoded by A. baumannii K loci are known to catalyze the same linkage, and, in general, they share modest levels of amino acid identity. For example, Wzy_KL128_ and Wzy_KL27_ form the same linkage but are only 53% identical (19). The alternate, phage-encoded Wzy_Ph1_ protein described here shares modest but significant levels of identity (30 to 40% identity) with the Wzy proteins encoded by KL37 and KL116, as well as KL3, KL22 and KL52, and the same β-d-Gal*p*NAc-(1→3)-d-Gal*p* linkage is found between K127 units when Wzy_Ph1_ is present and in K37 and K116 (Fig. 3) as well as K3, K22 and K52 CPS (Table 2). This provided strong initial support for the proposal that the phage-encoded Wzy_Ph1_ is the functional Wzy polymerase for the CPS produced by A. baumannii 36-1454, and, in the future, predictions of the linkage formed may be able to be based on these lower levels of identity.

Roles for genes carried by bacteriophage in the modification of surface polysaccharide structures have been observed in several bacterial species, including A. baumannii. Previously, we found acetyltransferase genes in prophage sequences integrated into the genomes of different A. baumannii isolates and demonstrated that the CPS structures were 4-O-acetylated when the acetyltransferase gene was present (16). Knowledge of these genetic determinants and their influence on the structural makeup of A. baumannii CPS is critical to build our understanding of this important surface structure in order to facilitate its use as a therapeutic target to control this globally significant pathogen. Hence, better methods will be needed to find additional *wzy* genes that may be changing the CPS configuration in order to accurately identify the CPS from genome sequences.

## MATERIALS AND METHODS

### Bacterial strain, cultivation, and isolation of CPS.

A. baumannii 36-1454 was obtained from the collection of multidrug-resistant and extensively drug-resistant A. baumannii isolates of the Institute of Antimicrobial Chemotherapy, Smolensk State Medical University (Smolensk, Russia). Bacteria were cultivated in 2× TY medium overnight; cells were harvested by centrifugation (10,000 × *g*, 15 min) and suspended in phosphate-buffered saline (PBS), and 2 volumes of acetone was added to the suspension. Cells were spun down by centrifugation (10,000 × *g*, 15 min), and the precipitant was dried. After intense shaking, the precipitate (CPS) was separated by centrifugation (8,000 × *g*, 20 min) and dissolved in water, the pH value was adjusted to pH 8 by adding 1 M NaOH, and the CPS was precipitated with acetone and separated by centrifugation as described above, dissolved in distilled water, and applied to a column (53 by 3.5 cm) of Sephadex G-50 Superfine (Amersham Biosciences, Sweden). Elution was performed with 0.1% acetic acid (HOAc) and monitored using a UV detector (Uvicord, Sweden) at 206 nm. Purified CPS samples were obtained in yields of 20 to 40 mg.

### Chemical analyses.

A CPS sample (1 mg) was hydrolyzed with 3 M CF_3_CO_2_H (120°C, 2 h). Monosaccharides were analyzed using a Biotronik LC-200 sugar analyzer. Neutral sugars were identified on a column (15 by 0.4 cm) of Dionex Ax8 anion-exchange resin in 0.5 M sodium borate buffer, pH 8, at 70°C. Amino sugars were determined on a column (22 by 0.4 cm) of Ostion LC AN B cation-exchange resin in 0.2 M borate buffer pH 5 at 70°C.

### Smith degradation.

A sample of the CPS from strain 36-1454 (12 mg) was oxidized with aqueous 0.05 M NaIO_4_ (1.6 mL) at 20°C for 40 h in the dark and reduced with NaBH_4_ (48 mg) at 20°C for 16 h. The excess NaBH_4_ was destroyed with concentrated HOAc, the solution was evaporated, methanol was added to the residue (3 × 1 mL) and evaporated, and the residue was dissolved in 0.3 mL water and applied to a column (35 by 2 cm) of Sephadex G-50. The modified polysaccharide was eluted with aqueous 0.1% HOAc and hydrolyzed with 2% CH_3_CO_2_H (100°C, 2 h). Fractionation of the products by gel permeation chromatography on a column (108 by 1.2 cm) of Sephadex G-25 in water gave an oligosaccharide (2.4 mg).

### NMR spectroscopy.

Samples were deuterium exchanged by freeze-drying from 99.9% D_2_O and then examined as solutions in 99.95% D_2_O. NMR spectra were recorded on a Bruker Avance II 600-MHz spectrometer (Germany) at 60°C. Sodium 3-trimethylsilylpropanoate-2,2,3,3-d_4_ (δ_H_ 0, δ_C_ −1.6) was used as an internal reference for calibration. Two-dimensional NMR spectra were obtained using standard Bruker software, and the Bruker TopSpin 2.1 program was used to acquire and process the NMR data. A 60-ms MLEV-17 spin-lock time and a 150-ms mixing time were used in ^1^H,^1^H TOCSY and ROESY experiments, respectively. A 60-ms delay was used for evolution of long-range couplings to optimize ^1^H,^13^C HMBC experiments for the *J*_H,C_ coupling constant of 8 Hz.

### Sequencing and bioinformatic analysis.

The genomes of 36-1454 and KZ-1257 were sequenced on a MiSeq platform using a Nextera DNA library preparation kit (Illumina, San Diego, CA), and the reads were assembled into contigs using SPAdes v3.10 (32). The draft genome sequences were deposited in NCBI under accession numbers JAHTLH000000000.1 for 36-1454 and JALDNC000000000 for KZ-1257. Coding sequences were translated and annotated using Prokka v1.14.15 (33). The sequence of the CPS biosynthesis gene cluster was extracted and annotated according to the established nomenclature system (9, 10). The annotated sequence was deposited in NCBI GenBank under accession number MK399427.1.

Whole-genome sequences for strains with determined CPS structures examined in this study were downloaded from NCBI (ATCC 17978, K3, GenBank accession number CP012004.1; and NIPH146, K37, GenBank accession number APOU01000001.1). The number of transmembrane segments for each coding sequence was predicted using TMHMM v2.0 (https://services.healthtech.dtu.dk/service.php?TMHMM-2.0), and protein families were identified using hmmscan v.2.41.2 (34). Phage sequences were identified and characterized using PHASTER (35). Pairwise sequence alignments and percentage identity matrices to assess relationships were constructed using CLUSTAL Omega (https://www.ebi.ac.uk/Tools/msa/clustalo/) and visually using Easyfig (36).

### Data availability.

Whole-genome sequence data from this study are deposited in NCBI under accession numbers JAHTLH000000000.1 (36-1454) and JAJAWC000000000.1 (KZ-1093).
